# Isolated Systolic Hypertension and its Determinants - A Cross-sectional Study in the Adult Population of Lucknow District in North India

**DOI:** 10.4103/0970-0218.62579

**Published:** 2010-01

**Authors:** Tanu Midha, MZ Idris, RK Saran, AK Srivastava, SK Singh

**Affiliations:** Department of Community Medicine (SPM), GSVM Medical College, Kanpur, India; 1 Department of Upgraded Department of Community Medicine (SPM), King George Medical University, Lucknow, India; 2 Department of Cardiology, King George Medical University, Lucknow, India

**Keywords:** Age, body mass index, isolated systolic hypertension

## Abstract

**Objectives:**

1) To determine the prevalence of isolated systolic hypertension (ISH) in the adult population of Lucknow district. 2) To study the determinants of ISH especially the relationship with age.

**Materials and Methods:**

A community-based cross-sectional study was conducted in four randomly selected areas of Lucknow district. A total of 800 subjects, aged 20 years and above, 400 from urban and 400 from rural area of Lucknow district were included in the study. The statistical tools used for analysis were Pearson's Chi-square test and multiple logistic regression.

**Results:**

The prevalence of ISH according to JNC-7 criteria was 4.3%, which was 5.1% in men and 3.6% in women. A significant increase in the prevalence of ISH was seen with an increase in age. Multivariate logistic regression analysis of the determinants showed that age, BMI and smoking were significant independent risk factors of ISH.

**Conclusions:**

Given the risk of cardiovascular disease associated with ISH, the findings of this study emphasize the need for further research to document the impact of modifiable risk factors of ISH and the effect of hypertension screening and specific health promotion in bringing down the burden of ISH and related cardiovascular morbidity.

## Introduction

Isolated systolic hypertension (ISH), defined as a raised systolic pressure but normal diastolic pressure, was originally considered part of aging and, like essential hypertension, benign.(1) However, there is now compelling evidence from cross-sectional, longitudinal and randomized controlled trials that show that ISH confers a substantial cardiovascular risk. One of the key messages of the Seventh Report of the Joint National Committee (JNC-7) on prevention, detection, evaluation and treatment of high blood pressure(2) is that in those older than age 50 years, systolic blood pressure of greater than 140 mmHg, is a more important cardiovascular disease (CVD) risk factor than diastolic blood pressure. Isolated systolic hypertension leads to a two-fold increase in risk of cardiovascular accidents as well as a similar rise in the risk of myocardial infarction.(3) Prevention and treatment of ISH leads to lowering of cardiovascular morbidity and mortality.(4) However, reports on the prevalence of ISH in developing countries like India are very scarce, therefore the present study was undertaken to estimate the prevalence of ISH based on the recently formulated criteria of the JNC-7(5) and to identify the determinants of the same.

## Materials and Methods

The study was designed to ascertain the prevalence of hypertension in the adult population (≥20 years of age) of Lucknow district, a city in north India and the capital of Uttar Pradesh, the largest state in the country. The prevalence of hypertension in India is reported as ranging from 10 to 30.9%, which is, on an average about 20%.(6) The sample size required was calculated according to an estimated prevalence of hypertension (*p*) of 20%, with a relative precision (*d*) of 20% of prevalence and a confidence level of 95%, using the formula:(7) Z(1-α/2)^2^ *pq/d*^2^. The sample size thus obtained was 400. Since it was a multistage stratified random study, a design effect of 2 was taken to reduce any inherent variation. The final sample size was therefore taken as 800. The study was conducted from August 2003 to July 2004.

Multistage stratified random sampling technique was used to select the study sample. In the first stage, Lucknow district was divided into rural and urban areas and 400 study subjects were included from each of these areas. In the secongd stage, two wards, one cis-Gomti and one trans-Gomti (Gomti river divides urban Lucknow, geographically) were randomly selected out of 110 wards in urban area and 2 PHCs were randomly selected out of 14 PHCs in rural area. In the third stage, two areas (one slum and one non-slum) were randomly selected from each ward in urban area and two villages (one subcenter village and one non-subcenter village) were randomly selected from each PHC in rural area. Systematic random sampling technique was used to select 100 subjects from each village and from each non-slum and slum area making a total of 400 subjects from rural and urban area, respectively.

Informed verbal consent was taken from each of the participants. A pre-tested structured questionnaire was used to elicit the information regarding the socio-demographic characteristics and the level of physical activity of the study participants. Social class was calculated using modified Kuppuswamy(8) scale in urban area and Pareek(9) scale in rural area. Classes 1, 2, 3 together were considered as upper class and classes 4 and 5 as lower class. Physical activity can be expressed as increments of BMR.(10) In the present study, the subject's BMR factor(11) was calculated by questioning him/her about the type of activity and time spent in performing each activity in the last 24 h. On the basis of the BMR factor, the level of activity was classified as follows:

Average daily level of activity of adults classified as sedentary, moderate or heavy, expressed as a multiple of BMR, is as follows:

**Table:** 

Sex	Sedentary	Moderate	Heavy
Men	1.55	1.78	2.10
Women	1.55	1.64	1.82

A smoker was defined as a subject who smoked any number of cigarettes daily, and/or one who had left smoking less than one year back. A non-smoker was defined as one who had never smoked in his lifetime or one who had left smoking at least one year back. Similarly an alcoholic was defined as a subject who consumed any amount of alcohol at least once in a week and/or one who had left alcohol consumption less than one year back. On the other hand, a non-alcoholic was defined as one who had never consumed alcohol in his lifetime or one who had left alcohol consumption at least one year back. The salt intake was calculated by asking for the average monthly consumption of salt by the whole family and dividing it by the number of persons in the family and then dividing it by 30 to calculate the average daily intake in g/person/day. JNC-7(5) has recommended a daily intake of salt of no more than 100 mEq/L, which is equivalent to 6 g/day of sodium chloride or 2.4 g/day of sodium. Therefore, the association of ISH with salt intake was analysed after dividing the subjects into those consuming <6, 6-8 and >8 g/day.

According to JNC-7, ISH was defined as a systolic blood pressure (SBP) ≥140 mmHg and a diastolic blood pressure (DBP) <90 mmHg. A standard mercury sphygmomanometer (Diamond Co.) was used for recording blood pressure. Before the measurement was taken, the subject was seated comfortably for at least 5 min. Care was taken that arm muscles were relaxed and the arm was supported at heart level. The cuff was applied evenly to the upper arm and was rapidly inflated until the manometer reading was 30 mmHg above the level at which the radial pulse disappears, and then slowly deflated at the rate of approximately 2 mmHg/s. During this time, the Korotkoff sounds were monitored using a stethoscope placed over the brachial artery. The first (appearance) and the fifth (disappearance) Korotkoff sounds were recorded as indicative of SBP and DBP, respectively. Systolic and diastolic blood pressures were measured twice and the mean for each was calculated.

Body weight was measured, to the nearest 0.5 kg, using a standard Krup's weighing machine, with the subject standing motionless on the weighing scale, feet about 15 cm apart, and weight equally distributed on each leg. Height was measured, to the nearest 0.5 cm, with the subject in an erect position against a vertical surface, and with the head positioned so that the top of the external auditory meatus was at level with the inferior margin of the bony orbit.

Data was analysed using the software SPSS 10.0.1 for Windows. The prevalence rates were given as percentages. Discrete data was analysed using Pearson's Chi-square test. In case the expected values in some cells were <5, Fischer's Exact test was used in place of Chi-square test. The significance of various risk factors was calculated by multivariate logistic regression analysis in which the Odd's ratio and 95% confidence intervals were calculated. In the multivariate model, the presence of ISH was taken as the dependent variable and age, place of residence, physical activity, body mass index, smoking and alcohol were taken as independent variables. Two-tailed *P* values less than 0.05 were considered significant.

## Results

A total of 800 subjects were studied, 400 in urban and 400 in rural area of Lucknow district. The study sample comprised of 445 women (55.6%) and 355 men (44.4%). The prevalence of hypertension in the study population was found to be 23.3% (95% CI: 20.4-26.2%), whereas that of ISH was 4.3% (95% CI: 2.9-5.7%). Prevalence of ISH was 5.1% (95% CI: 3.6-6.6%) in men and 3.6% (95% CI: 2.3-4.9%) in women and this difference was not statistically significant (*P* = 0.287). In the present study, 43.0% of the total hypertensives were found to be aware of their hypertensive status and 26.5% of the subjects with ISH were aware of their condition.

For the analysis of association of ISH with various determinants, we compared subjects having ISH (*n* = 34) with the normotensives (*n* = 614). The rest of the 152 hypertensives with either diastolic hypertension alone or both systolic and diastolic hypertension were not included in the analysis to eliminate any bias resulting thereof due to their association with the risk factors. Therefore a total of 648 subjects were included in the analysis to study the association between various risk factors and ISH.

The study revealed a significant association of ISH with age (*P* < 0.001) as shown in Table 1. Prevalence was found to be high in ≥60 years' age group (45.8%) and low in 20-29 years age group (1.0%). Prevalence was 2.2% in 30-39 years age group followed by 12.5% in 40-49 years age group and 13.6% among subjects 50-59 years old.

**Table 1:** Determinants of isolated systolic hypertension

Determinants	ISH (*n* = 34)	Total (*n* = 648)	% of ISH
Age (years)			
20-29	3	315	1.0
30-39	4	185	2.2
40-49	10	80	12.5
50-59	6	44	13.6
≥60	11	24	45.8
*P* = 0.000*			
Place of residence			
Urban	18	292	6.2
Rural	16	356	4.5
*P* = 0.343			
Socioeconomic class			
Upper	5	59	8.5
Lower	29	589	4.9
*P* = 0.244			
Family type			
Nuclear	19	467	4.1
Joint	15	181	8.3
*P* = 0.031*			
Religion			
Hindu	21	462	4.5
Others$	13	186	6.9
*P* = 0.206			
Marital status			
Married	25	528	4.7
Others#	9	120	7.5
*P* = 0.213			

* *P* values < 0.05 are significant.

$ Muslim, Sikh, Christian.

# Unmarried, divorced, widowed

A significant association of ISH was observed with the type of family (*P* = 0.031) [Table 2]. The prevalence of ISH increased with decreasing level of physical activity (*P* = 0.01). The lowest prevalence of ISH was found among those with heavy level of physical activity (0.0%), and the highest prevalence was seen among those with a sedentary lifestyle (7.3%). There was a significant increase (*P* = 0.006) in the prevalence of ISH with higher BMI. Prevalence was 2.8% among subjects with BMI <18.5 kg/m^2^ and 21.1% among subjects with BMI ≥30 kg/m^2^. A significant difference in the prevalence of ISH was seen between smokers (9.6%) and non-smokers (3.3%), and between alcoholics (22.2%) and non-alcoholics (4.8%).

**Table 2:** Lifestyle-related determinants of isolated systolic hypertension

Determinants	ISH (*n* = 34)	Total (*n* = 648)	% of ISH
Physical activity			
Sedentary	28	381	7.3
Moderate	6	197	3.0
Heavy	0	70	0.0
*P* = 0.010*			
Body mass index (kg/m^2^)			
<18.5	5	177	2.8
18.5-24.9	18	359	5.0
25-29.9	7	93	7.5
≥30	4	19	21.1
*P* = 0.006*			
Smoking			
Non-smoker	15	450	3.3
Smoker	19	198	9.6
*P* = 0.001*			
Alcohol			
Non-drinker	30	630	4.8
Drinker	4	18	22.2
*P* = 0.006*			
Salt Intake (g/day)			
<6	5	104	4.8
6-8	2	140	1.4
>8	27	404	6.7
*P* = 0.055			

* *P* values < 0.05 are significant.

On the other hand, although the prevalence of ISH was higher in people residing in urban areas (6.2%) as compared to that in rural areas (4.5%), this difference was not statistically significant (*P* = 0.343). Similarly the differences observed in the subjects belonging to the upper socioeconomic class (8.5%) as compared to the lower socioeconomic class (4.9%) were also due to chance alone (*P* = 0.244). The prevalence of ISH did not differ significantly among the different religious groups (*P* = 0.206).

Multivariate logistic regression analysis revealed that age (OR = 2.733), BMI (OR = 2.204) and smoking status (OR = 2.709) were significant independent predictors of ISH [Table 3], whereas place of residence, physical activity and alcohol did not affect the prevalence of ISH.

**Table 3:** Multivariate logistic regression analysis for association of various determinants with prevalence of isolated systolic hypertension

Determinants	Odd's ratio	95% confidence interval		*P* value
			
		Lower limit	Upper limit	
Constant				0.000
Age				
(0 = 20-30 years, 1 = 30-40 years, 2 = 40-50 years, 3 = 50-60 years, 4 ≥ 60 years)	2.733	1.979	3.776	<0.000*
Place of residence				
(0 = Urban, 1 = Rural)	1.073	0.470	2.452	0.867
Physical activity				
(0 = sedentary, 1 = moderate, 2 = heavy)	0.429	0.184	1.001	0.050
BMI				
(0 ≤ 18.5, 1 ≥ 18.5 and < 25, 2 ≥ 25 and < 30, 3 ≥ 30)	2.204	1.318	3.686	0.003*
Smoking				
(0 = non-smoker, 1 = smoker)	2.709	1.158	6.337	0.022*
Alcohol				
(0 = non-alcoholic, 1 = alcoholic)	2.632	0.581	11.936	0.209

* *P* values, < 0.05 are significant. The reference category is: Normotensive

## Discussion

This study shows that the prevalence of ISH was 4.3% (5.1% in men and 3.6% in women), which was lower than that reported among office workers in the north Indian town of Shimla (7.8% overall, 7.9% in men and 6.7% in women).(12) This difference may be explained by the fact that the economically productive population is prone to job-related stress, which can adversely affect the prevalence and is a possible source of confounding if the results are compared with the general population. The prevalence was lower in women in the Shimla study, similar to the finding observed in our study. In the Chinese adult population,(13) the prevalence of ISH was observed to be 7.6%. In Delhi,(14) the prevalence of ISH was observed to be 15.3% in subjects aged 60 years and above.

The differences observed could be due to the discrepancy in the sample size and sampling technique. The sample size in the present study was calculated to estimate the prevalence of hypertension in the adult population of Lucknow district, therefore it may not be appropriate to determine the prevalence of ISH. Having selected an equal number of subjects (100) from each area from a non-PPS (Probability Proportion to Size) distribution does not give appropriate population weight and may not be reflective of the actual population distribution. This study does not draw conclusive evidence regarding the prevalence but does highlight the fact that further studies with a larger sample size need to be done in order to obtain realistic estimates of the burden of ISH, which remains underdiagnosed and largely untreated. The roots of this lie in a century of overreliance on the importance of diastolic pressure and unjustified concerns about the potential adverse consequences of treating systolic pressure.(1)

A significant association of ISH was seen with increasing age, an observation similar to that reported in the Chinese adult population.(13) The prevalence of ISH was significantly associated with physical activity, BMI and alcohol intake as also observed in the Shimla study. However, there was no association between ISH and salt intake as seen in the Shimla study. This may be because the quantification of salt intake is prone to subjective errors. Actual measurement of daily salt intake is required for assessment of its effect on hypertension. Secondly, the daily average was estimated by dividing the total family intake by the number of family members, which can only be considered as a proxy for consumption unit. Extra salt intake from other sources like pickles and fast foods, having high salt content, has also not been accounted for.

The association of ISH with age, smoking, alcohol intake and BMI was similar to that seen in another study from China,(15) although in the present study there was no association with gender, religion and salt intake as reported in the Chinese adult population. The prevalence of ISH in association with level of physical activity, BMI, smoking and alcoholism was according to the expectations and further substantiates previous researches.

Physical activity, assessed in this study as increments of BMR, takes into account only the activities of the past 24 h, which might have biased the estimate of level of physical activity. ‘Non-smoker’ and ‘non-alcoholic’ has been defined taking a cut off of the past one year, irrespective of the total duration of smoking and alcohol consumption, respectively. This could have led to a misclassification bias and dilution of the observed effect on hypertension. Smokeless tobacco has not been considered in this study, but an association with hypertension needs to be explored. Being a cross-sectional study, the possibility of bias due to lifestyle modification in subjects aware of their hypertensive status cannot be overruled. In the present study, 26.5% subjects with ISH were aware of their hypertensive status. The study design is not a very good one to look at determinants, nevertheless it highlights the fact that ISH is not a purely age-related phenomenon and further research is required to identify other factors influencing the prevalence of ISH and to elucidate measures for its control.

Of all these factors, increasing age, higher BMI values and smoking were significant independent predictors of ISH as revealed in multivariate logistic regression analysis. This emphasizes the need for further research to document the impact of modifiable risk factors of ISH and the effect of primary prevention in bringing down the burden of ISH. Given the risk of cardiovascular disease associated with ISH, hypertension screening and health education programs regarding weight reduction and cessation of smoking may be considered as a cost-effective public health approach in dealing with the morbidity attributed to ISH and cardiovascular diseases. This is a cross-sectional study designed to formulate the hypothesis for future research. This study is only a prelude to the upcoming research in the field of non-communicable diseases and indicates the insidious presence of ISH in the adult population and the underlying factors, which may be responsible for its occurrence.
